# Aflatoxins: History, Significant Milestones, Recent Data on Their Toxicity and Ways to Mitigation

**DOI:** 10.3390/toxins13060399

**Published:** 2021-06-03

**Authors:** Darina Pickova, Vladimir Ostry, Jakub Toman, Frantisek Malir

**Affiliations:** 1 Department of Biology, Faculty of Science, University of Hradec Kralove, Rokitanskeho 62, CZ-50003 Hradec Kralove, Czech Republic; ostry@chpr.szu.cz (V.O.); jakub.toman@uhk.cz (J.T.); frantisek.malir@uhk.cz (F.M.); 2 Center for Health, Nutrition and Food in Brno, National Institute of Public Health in Prague, Palackeho 3a, CZ-61242 Brno, Czech Republic

**Keywords:** turkey “X“ disease, aflatoxin, milestones, toxicity, mitigation

## Abstract

In the early 1960s the discovery of aflatoxins began when a total of 100,000 turkey poults died by hitherto unknown turkey “X” disease in England. The disease was associated with Brazilian groundnut meal affected by *Aspergillus flavus*. The toxin was named *Aspergillus flavus* toxin—aflatoxin. From the point of view of agriculture, aflatoxins show the utmost importance. Until now, a total of 20 aflatoxins have been described, with B_1_, B_2_, G_1_, and G_2_ aflatoxins being the most significant. Contamination by aflatoxins is a global health problem. Aflatoxins pose acutely toxic, teratogenic, immunosuppressive, carcinogenic, and teratogenic effects. Besides food insecurity and human health, aflatoxins affect humanity at different levels, such as social, economical, and political. Great emphasis is placed on aflatoxin mitigation using biocontrol methods. Thus, this review is focused on aflatoxins in terms of historical development, the principal milestones of aflatoxin research, and recent data on their toxicity and different ways of mitigation.

## 1. Introduction

In 2020, it was 60 years since the discovery of aflatoxins (AFs). AFs began the “second mycotoxicology era” that built on the “previous mycotoxicology era”, e.g., ergotism, acute cardiac beriberi, alimentary toxic aleukia, stachybotriotoxicosis, “mouldy corn toxicosis”—equine leucoencephalomalacia [1,2,3,4].

## 2. A History of Aflatoxin Discovery

In the late 1950s and early 1960s, a new so far unknown turkey disease, characterized by heavy mortality, was identified in England. After the turkey disease outbreak of unknown nature and aetiology (the turkey “X” disease), the discovery of AFs began. A total of 100,000 turkeys died of so-called turkey “X” disease after being fed with contaminated Brazilian groundnut meal on a poultry farm in London [5].

**Figure 1:**
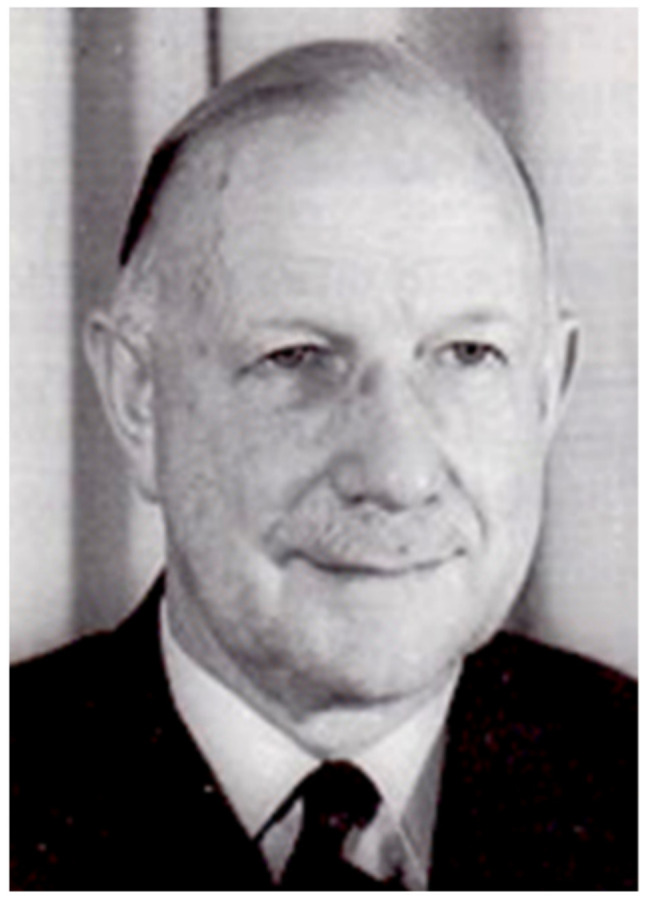
William Percy Blount (*1905–^+^1968). Photo: World’s Poultry Science Association.

William Percy Blount (Figure 1) was a veterinary scientist and consultant in poultry husbandry and developed a highly effective poultry disease diagnostic service for the customers of a major feed compounding company in England.

Chief poultry advisor W.P. Blount carried out intensive field and laboratory research, the findings and conclusions of which were published the following year [5]. Many affected turkeys were about 4–6 weeks old, but others were aged 10–16 weeks. Affected turkeys naturally sought the heat of their brooders and then, through weakness, they would sink to the floor and become somnolent, death taking place within 24–48 h. Another characteristic of the disease was the position or the posture adopted by the poults when they died. Mortality varied but was usually high, often with death rates of 50–90% [5]. By the time the disease had subsided, about 500 outbreaks had been reported involving an estimated loss of over 200,000 turkeys [6].

A causal relationship between feed toxicity, Brazilian groundnut meal, and disease has been demonstrated by W.P. Blount, who accurately described the symptoms, especially liver lesions, and subsequently excluded that the disease could be the cause of the infectious agents. The turkey “X” disease had to be differentiated from Non-specific enteritis, Transmissible enteritis, Infectious hepatitis, Newcastle disease, New “virus” infection, and “poisoning” (bacterial, fungal, mineral, vegetable, etc.). Because veterinary examinations for pathogenic microorganisms were generally negative, and as the epidemiological picture was not that which might be associated with an “infection”, the final possibility that the turkeys were “poisoned” remained. The histological findings, post mortem in young turkeys that had died suddenly were described in detail in other studies. They also suggested that the disorder was caused by poisoning, but the toxic substance had not been identified [7,8].

W.P. Blount had also excluded the participation of chemical agents and potentially toxic chemicals that are commonly found in poultry feed, either as contaminants (e.g., toxic elements, pesticides, glycosides, alkaloids, natural phytotoxins, etc.), ingredients (a toxic compound in Brazilian groundnut meal), or as a result of dishonest practices. Although his efforts did not lead to the identification of a causative factor, he provided a solid basis for peer scientists to make relatively rapid progress towards the goal [5].

The disease continued in the same area of London and induced deaths among turkeys on farms as a result of previous feeding with Brazilian groundnut from mills carried by the same company, which included groundnut in the composition of the feed. Groundnut meal was later proved as the main suspect [6,9,10,11]. Similar liver lesions, considered to be the most serious damage, were found during post mortem examination tests on ducklings, chicken, young pheasants, cattle, rats, and pigs fed with Brazilian groundnut meal [6,9,10,12,13].

**Figure 2:**
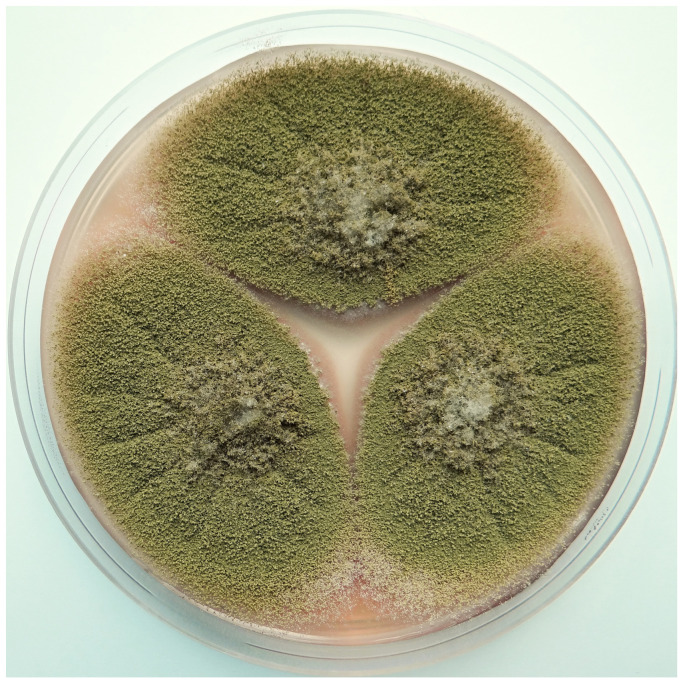
Macroscopic view of *Aspergillus flavus* on Czapek yeast extract agar (25 °C, 7 days). Photo: Vladimir Ostry.

**Figure 3:**
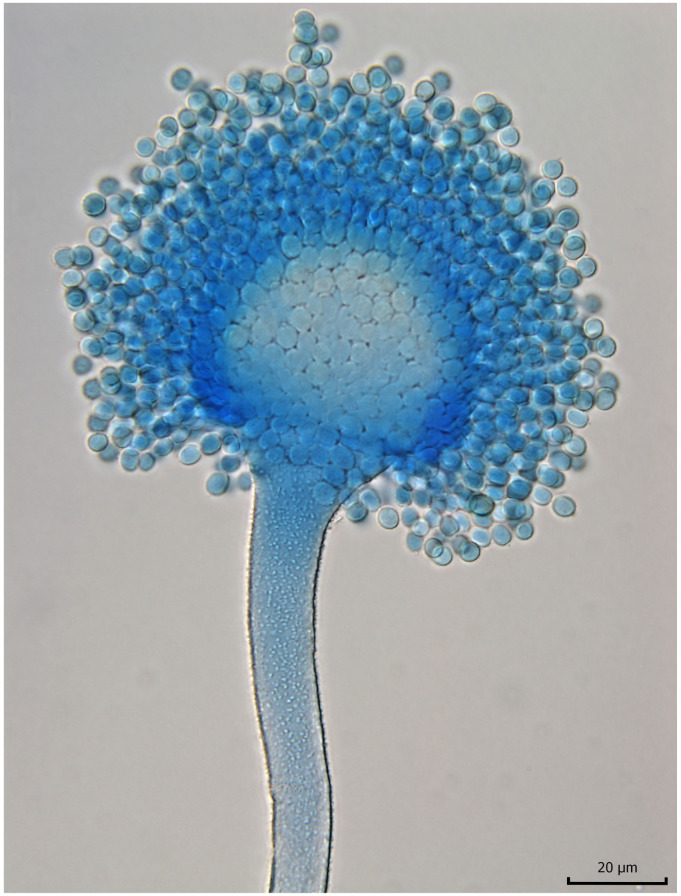
Light microscopy of *Aspergillus flavus* with lactophenol cotton blue. Photo: Vladimir Ostry.

The rats fed on groundnut meal showed toxic effects with the development of liver cancer [14,15], which was later confirmed by several studies [16,17,18,19]. An investigation of the peanut meal determined that it was highly toxic, with a naturally occurring toxic metabolite produced by mold infestations that caused the acute toxic effects in the animals [9]. Although it had been suspected since the end of 1960 that the cause might be a toxin [10], this was not finally established until the end of the following year when it was demonstrated that metabolites synthesized by some strains of *Aspergillus flavus* Link ex Fries were responsible [20]. Mold contaminant of *Aspergillus flavus* Link ex Fries was identified in the Central Veterinary Laboratory at Weybridge in England. A replica of *A. flavus* identification is shown in Figure 2 and Figure 3.

Selected isolates of *A. flavus* were cultivated on mycological media under laboratory conditions. The cultures with overgrown mycelium of *A. flavus* were extracted with CHCl_3_. Paper chromatography was used in this experiment with the mobile phase butan-1-ol: CH_3_COOH. A total of 12.5% of tested extracts emitted a blue fluorescence spot (retention factor (Rf) value of 0.7) under UV light. After oral administration of the corresponding extract to one-day-old ducklings, death was observed within 24 h due to typical symptoms of turkey “X” disease, especially liver damage [10].

The isolation of the toxin crystalline form responsible for the turkey “X” disease has been performed [21]. Because the extract was poorly pure, the blue fluorescent spot probably contained more toxic metabolites. After separation and quantification by thin layer chromatography, two different fluorescent spots were detected, the former emitting blue (Rf 0.6) and the latter emitting green (at slightly lower Rf) fluorescence. *Aspergillus flavus* toxin gave rise to the name aflatoxin. The name aflatoxin was given to the toxic substance, which has since been found to contain several closely related toxic components.

AF discovery was a collective effort, involving a number of experts from various fields of research in veterinary medicine, animal nutrition, toxicology, chemistry, and mycology, and etc.

## 3. The Milestones in Aflatoxin Research

Research on AFs in all areas of interest is very extensive. Several valuable reviews on AFs have been published in the last decade [1,4,22,23,24,25,26,27,28,29,30].

These primary and valuable articles served to prepare the principal milestones of AF research. The older data from the years 1960 to 1990 were independently confirmed. Studying the principal milestones allows us to present an overview from several fields of research on AFs from their discovery to 2021.

**Figure 4:**
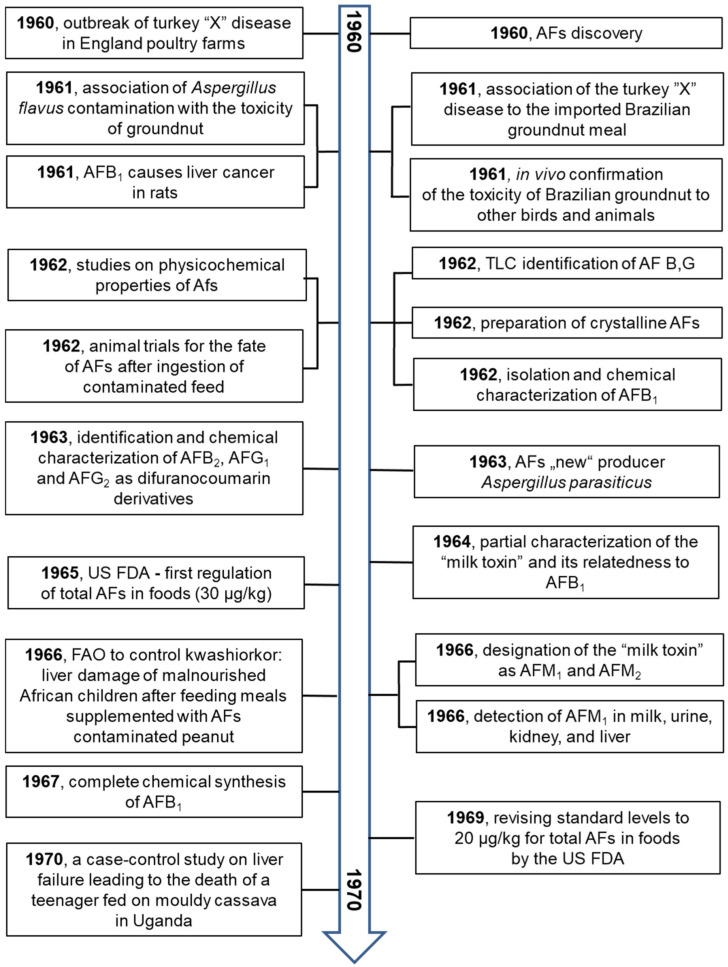
The milestones in aflatoxin research over the years 1960–1970 [5,9,11,12,13,14,15,17,19,20,21,31,32,33,34,35,36,37,38,39,40,41,42,43,44,45,46,47,48].

**Figure 5:**
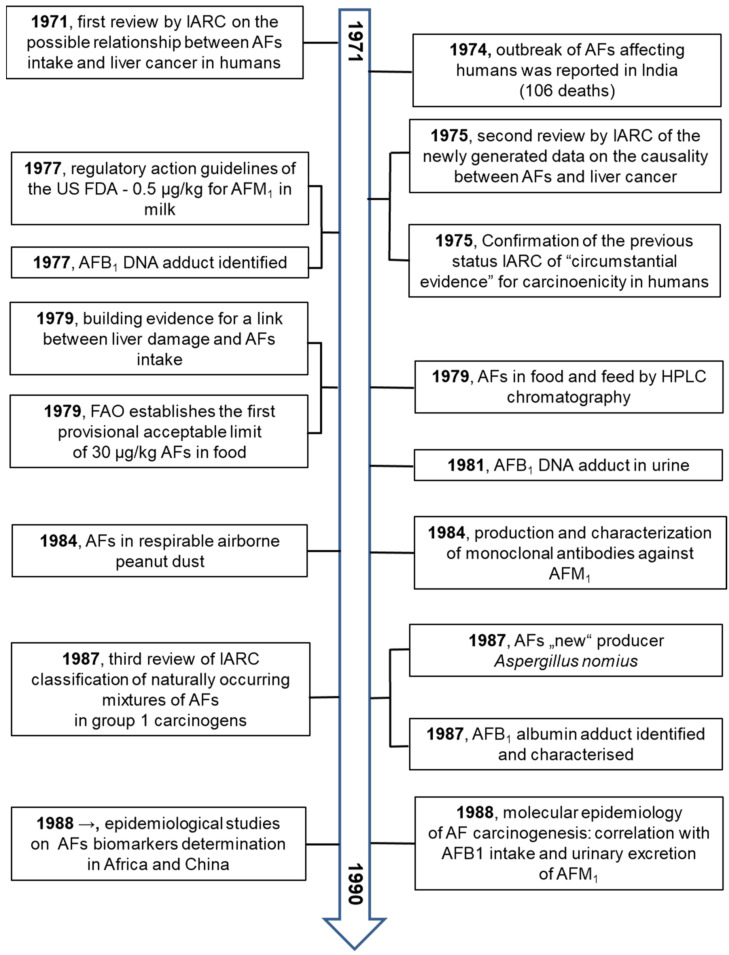
The milestones in aflatoxin research over the years 1971–1990 [49,50,51,52,53,54,55,56,57,58,59,60,61,62,63,64,65].

**Figure 6:**
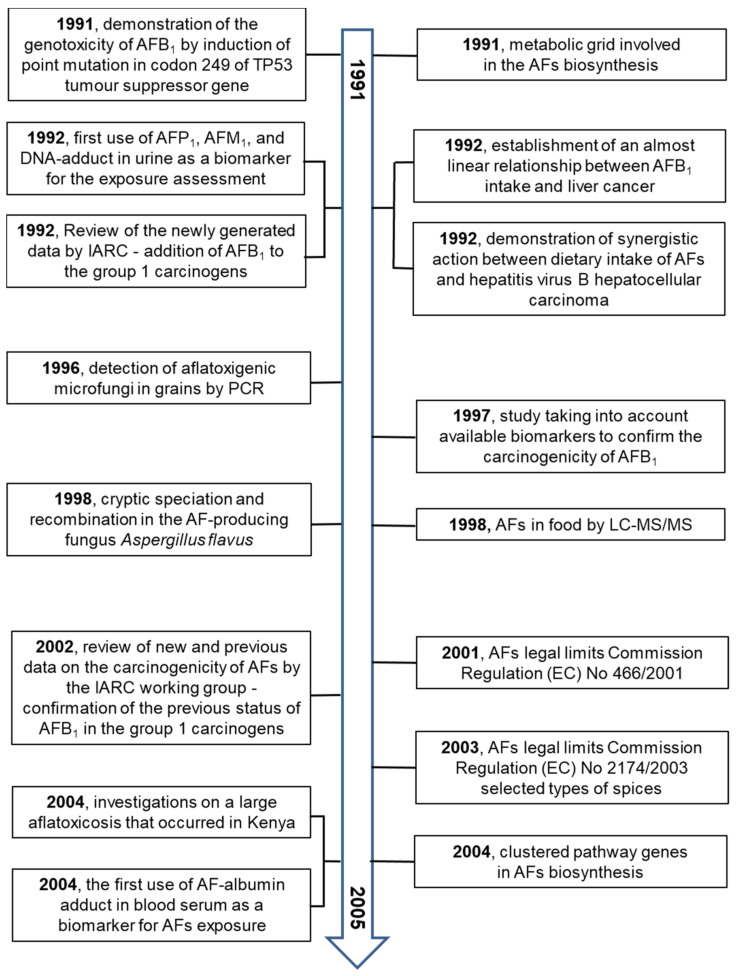
The milestones in aflatoxin research over the years 1991–2005 [66,67,68,69,70,71,72,73,74,75,76,77,78].

**Figure 7:**
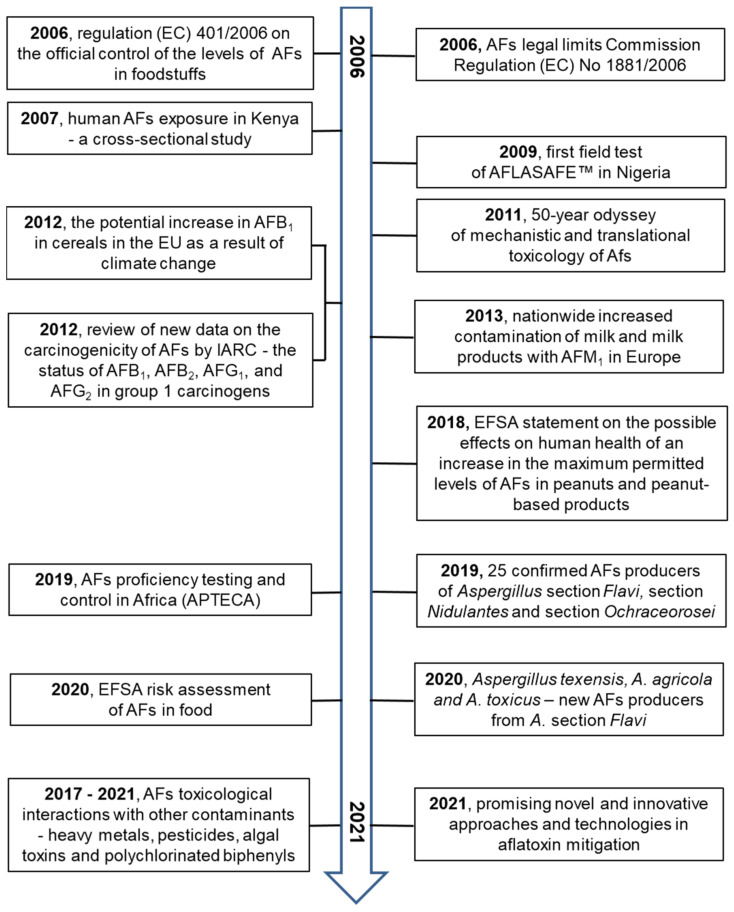
The milestones in aflatoxin research over the years 2006–2021 [26,79,80,81,82,83,84,85,86,87,88,89,90,91,92,93,94,95,96,97,98].

The principal milestones are summarized in Figure 4, Figure 5, Figure 6 and Figure 7.

## 4. Recent Data on Aflatoxin Toxicity

The human population is often exposed to low AF levels due to the daily intake of various AF-contaminated products [99]. Approximately 4.5 billion people worldwide, mainly in developing countries, have been estimated to be chronically exposed to AFs via contaminated food [100,101]. Moreover, due to the difficulties in food management and the socio-economic difficulties caused by the ongoing coronavirus pandemic (COVID-19), an increase in the consumption of AF-contaminated foods can be expected [102]. In 2020 according to the RASFF (Rapid Alert System for Food and Feed) database, AFs were the most often notified in peanuts; dried figs; spices; rice; and various nuts such as hazelnuts, almonds, and pistachios [103]. However, in recent years, some of these food products have shown a relatively high concentration exceeding 1000 µg/kg [103], which may be related to the development of aflatoxicosis, which can lead to serious health problems, in particular damage to the liver and other organs, primary liver cancer, and even death [104].

AFs are infamous for their high toxicity, therefore their presence in food (and feed) is highly feared. Naturally occurring AFs (AFB_1_, AFB_2_, AFG_1_, and AFG_2_) act as strong carcinogens, thus they are assigned into Group 1 “carcinogenic to humans” by the International Agency for Research on Cancer (IARC) [84,105]. Apart from their carcinogenicity, they have been reported to have mainly hepatotoxic, genotoxic, mutagenic, teratogenic, immunosuppressive, nephrotoxic, and cytotoxic effects [106,107,108,109].

Some toxic effects of AFs have been observed in the most recent literature. Hepatotoxic effect of AFB_1_ has been demonstrated in vivo on mice [110,111], rats [112], rabbits [113], and broiler chickens [114]. The nephrotoxic effect of AFB_1_ has been reported in vivo on broiler chickens [114] and rats [112]. The neurotoxicity of AFB_1_ has been observed in vitro on human astrocytes and in vivo on a glial cell in zebrafish [115]. The immunosuppression of AFB_1_ has been demonstrated in vitro on swine alveolar macrophages [116]. Reproductive toxicity of AFB_1_ has been demonstrated in vivo on mice [110]. Pulmonary toxicity of AFB_1_ has been observed in vivo on male albino rats [117]. Gastrointestinal toxicity of AFB_1_ has been described in vivo on rats [118], pigs [119], and chickens [120]. The genotoxic effect has been observed in vivo on mice [110]. Cytotoxic and/or genotoxic effects of AFB_1_ have been reported in vitro on the leghorn male hepatoma (LMH) cell line [121], the liver hepatocellular carcinoma (HepG2) cell line [122], buffalo rat liver (BRL-3A) cells [123], bovine mammary epithelial (BME) cells [124], and the human keratinocyte (HaCaT) cell line [125]. Cytotoxicity on BME cells has also been observed in vitro in the case of AFM_1_ [124]. The embryotoxicity of AFB_1_ has been reported in vitro on bovine embryos [126].

It should be emphasized that the impact of AFs, as well as other mycotoxins or contaminants in general, on human health always depends on the toxicological properties of the agent, the individual properties of the consumer, and duration of exposure to the agent, and also the presence of other contaminants with which an interaction (e.g., synergistic) could occur [127].

### 4.1. Toxicological Interactions of Aflatoxins and Other Mycotoxins

Worldwide, food (and also feed) may be infested by more than one type of mold. Moreover, most molds can produce several mycotoxins simultaneously, as a result of which humans and animals may be exposed to a “cocktail of mycotoxins” in their diet [95,128,129]. However, the regulations worldwide do not take into account the combined effects of co-occurred mycotoxins [130]. Various interactions (synergistic, additive, antagonistic) have been reported, mainly between AFs and ochratoxin A (OTA), fumonisins, and trichothecenes [128]. AFs with fumonisins and AFs with OTA are among the most common mycotoxin combinations in cereals and cereal products [130].

For example, in the recent literature, the antagonistic effect on inducing cytotoxicity on the LMH cell line has been observed between AFB_1_ and OTA [121]. On the contrary, these two mycotoxins, also in combination with zearalenone (ZEA), acted synergistically in negatively affecting the milk production, blood metabolism, and immune function of Laoshan goats [131]. Synergistic interaction between AFB_1_ and OTA has also been observed in vitro on swine alveolar macrophages (3D4/21) [132]. A synergistic effect has also been reported between AFB_1_, deoxynivalenol (DON), and ZEA on human epithelial (Caco-2) cells [133]. However, reduced cytotoxicity of DON on the viability of MA-10 Leydig cells in vitro has been observed when combined with AFB_1_ [134]. A synergistic effect has also been observed within the AF group between AFB_1_ and AFM_1_ in compromising intestinal integrity in vivo on mice and in vitro on Caco-2 cells [135].

### 4.2. Toxicological Interactions of Aflatoxins with Other Contaminants

Humans (but also animals) can be exposed to many other environmental toxins along with mycotoxins, such as heavy metals and pesticides, but also algal toxins [95]. Considering that some mycotoxins and other environmental toxins may share the same target organ or tissue, the monitoring of their common combined effects pose a challenge for further research as these agents are usually studied individually rather than in combination [95]. However, there are several recent studies that address the interaction of AFs with other contaminants.

Heavy metals such as nickel, arsenic, lead, chromium, mercury, and cadmium share the main target organ, liver, with AFs [96], and it is therefore important to research their interactions. In an in vivo study, AFB_1_ and cadmium chloride showed an additive interaction in inducing acute oral toxicity in kunming mice [136]. A synergistic effect of AFB_1_ and sodium arsenite has been observed, including cytotoxicity in vitro in urinary bladder (HUC-PC) cells [137].

The co-contamination of agricultural crops with AFs (and also other mycotoxins) and pesticides (insecticides, fungicides, herbicides) has been reported [95]. Therefore, due to their co-occurrence, it is necessary to consider their combined toxic effects. Recently, an antagonistic interaction between AFB_1_ and the insecticide chlorpyrifos has been observed on HepG2 cells for cytotoxicity and genotoxicity [122]. In the case of AFB_1_ and insecticide *p,p’*-DDT, a dose-dependent interaction has been observed on MA-10 Leydig cells in vitro—additive at doses of 16 µM (5671.84 µg/L and 4996.36 µg/L of *p,p’*-DDT and AFB_1_, respectively) and 32 µM (11,343.68 µg/L and 9992.64 µg/L of *p,p’*-DDT and AFB_1_, respectively) and antagonistic at 64 µM (22,687.36 µg/L and 19,985.28 µg/L of *p,p’*-DDT and AFB_1_, respectively) [134].

Microcystin-LR (MC-LR), a toxin that is produced by cyanobacteria *Microcystis aeruginosa*, has been observed to possibly increase the liver damage risk in people suffering from hepatitis B simultaneously exposed to AFB_1_ [94]. Enhanced effects of AFB_1_ and MC-LR interaction on genotoxicity and cytotoxicity have been observed on the human liver cell line (HL7702) in vitro [138]. In contrast, a recent study points to the possibility of an antagonistic effect of low levels of MC-LR on AFB_1_-induced hepatocarcinogenicity on HL7702 cells in vitro through decreasing CYPA1A2 expression and AFB_1_-DNA adduct generation, while demonstrating that exposure to a combination of MC-LR and AFB_1_ may not worsen liver damage compared to exposure to AFB_1_ alone on six-week old male Sprague-Dawley rats in vivo [139]. Additionally, the combination of AFB_1_ and MC-LR predominantly exerted antagonistic effects in cytotoxicity to HepG2 and Madin-Darby bovine kidney epithelial (MDBK) cell lines in vitro [140].

Apart from the above-mentioned contaminants, AFs can interact with many other toxic substances from the environment. For example, polychlorinated biphenyls (PCB) may potentiate/enhance the genotoxicity effect of AFB_1_ in the human hepatocyte line (L-02 cell line) by enhancing CYP1A1, CYP1A2, and CYP3A4 expression [141].

### 4.3. Toxicological Interactions of Aflatoxins with Hepatitis B and C Virus in Relation to Carcinogenicity

AFs may induce a number of cancer types (liver, breast, lung, gallbladder, esophageal), the best known of which is liver cancer [142]. Liver injury and hepatocellular carcinoma (HCC), one of the major types of liver cancer, are considered the main toxic impact of AFB_1_ [99,107,143,144]. Worldwide, approximately 5–28% of HCC occurrences are attributed to AF exposure [145]. Globally, a total of 905,677 new cases (corresponding to a crude rate of 11.6 cases per 100,000 people) and 830,180 deaths (corresponding to a crude rate of 10.7 cases per 100,000 people) due to liver cancer in both sexes and all ages were estimated in 2020. Based on the total number of cases, liver cancer is ranked the 6th and 3rd cancer type in incidence and mortality, respectively, worldwide [146]. More than 80% of HCC cases come from developing countries [147]. HCC has become a serious health problem, especially in sub-Saharan African countries and countries of southeast Asia, and it is also increasing in Europe and the United States [148,149].

Dietary exposure to AFs is considered the second largest environmental risk factor for liver cancer development [148] after viral hepatitis B or C infections [150], which act synergistically with AFs [149]. Especially in developing countries, hepatitis B is considered the main risk factor for HCC; however, dietary exposure to AFs also plays a significant role in HCC etiology [151]. A recent study on Iranian patients suffering from hepatitis B or C has demonstrated the potential involvement of AFB_1_ exposure as a mean risk factor in the HCC etiology in these patients [152]. Chronic hepatitis B virus infection may induce cytochrome P450s, which is responsible for the metabolism of non-toxic AFB_1_ to AFB_1_-8,9-epoxide (AFBO) metabolite, with highly toxic and mutagenic effects [153]. AFBO attacks DNA through binding to the N7 position of guanine residues to produce a pro-mutagenic unstable DNA adduct AFB_1_-N7-Guanin. This DNA-adduct induces a specific transverse mutation G:C to T:A at codon 249 of the tumor suppressor gene p53, which is involved in cell cycle regulation [149,154,155,156]. This mutation is typical for HCC patients from regions of high AF-exposure [149,155].

## 5. Recent Data on Aflatoxin Mitigation

Several valuable original research articles [157,158,159] and reviews [97,98,160,161,162,163] on AF mitigation have been published over the last three years.

AF mitigation means reducing the health risk from the occurrence of AFs in foodstuffs. AF mitigation is key to food safety and nutrition and is any process used to reduce AF concentrations in foodstuffs [164].

AFs represent a threat to food safety worldwide because they are considered to be among the most prominent and dangerous toxins that can affect any part of the food chain from pre-harvest to food processing. Prevention and mitigation of AF contamination is critical to protect consumers from the adverse health effects associated with AFs [161,165].

AF contamination arises at multiple points in the food system, from the field to the home (where pest attack or poor drying techniques and inadequate crop storage allow the *A. flavus* to grow), and to the marketplace (where lack of quality control allows contaminated food to be sold). It is important to equip producers, traders, and consumers with knowledge that can help them manage this issue [157,165].

The weather conditions prior to harvest play a cardinal role in the risk of AF production, and the globalization of trade flows, as well as climate change, lead to the occurrence of unexpected AFs in unusual products [166,167].

AFs present a significant health hazard to consumers. With the potential to contaminate a range of common foods and feeds, such as grains (wheat, corn, barley, rice, and oats), nuts, cocoa, and milk, AFs present an ongoing challenge to food safety all along the food chain. The ideal way to mitigate their risk to food safety is to prevent these toxins from entering the food chain at all, and a number of pre-harvest strategies based on good agricultural practices can help [103,164,165].

Even with the best prevention strategies, however, AFs can end up in the food chain given that they are ubiquitous worldwide and that ever-changing environmental conditions preclude strict elimination [157,163].

At the present time, to avoid unfavorable AFs effects on public health, great attention is being given to prevention as well as to pre-harvest methods intended for *A. flavus* contamination reduction [157,162,163,166].

Numerous post-harvest methods to combat AFs are also required, such as emerging physical methods (e.g., non-thermal treatments as pulsed electric fields), interventions with chemical agents (e.g., adsorbents, acids, enzymes, and gases), interventions with microbiological agents (e.g., bacteria, yeast and microfungi), and genetic engineering technologies. These methods have been reported to be effective in mycotoxin diminution in food and feed [97,98,160,161,162].

### 5.1. The Selected Effective Pre-Harvest Method for Aflatoxin Mitigation

A total of 22, 4, and 2 species of the genus *Aspergillus* from the *Flavi*, *Nidulantes*, and *Ochraceorosei* sections produce AFs, respectively, with *A. flavus* of section *Flavi* being the most important and best known species [103].

*A. flavus* may be divided to the L and S morphotypes. The S morphotype produces a lot of small sclerotia (average diameter <400 μm), few conidia, and a regularly high amount of AFBs [168]. In contrast, the L morphotype can produce less numerous, larger sclerotia (average diameter >400 μm), a lot of conidia, and mutable amount of AFBs. There are also L morphotype genotypes that are not able to produce AFs (i.e., non-aflatoxigenic) due to inversions, deletions, or defects in at least one of the AF biosynthesis genes (a single mutation in the *pksA* (*aflC*) gene of its AF pathway), which bring in a premature stop codon and cause it to be defective [169,170]. As a potential biocontrol agent, found in a peanut fields in Georgia, there is another non-aflatoxigenic *A. flavus* strain (NRRL 21882), which demonstrated an important effect against native aflatoxigenic strains in laboratory tests and is commercially known as Afla-Guard^®^. The complete absence of the AF gene cluster is responsible for its inability to produce AFs [170]. Further, in Italy, the biopesticide (AF-X1TM) was evolved for protection of fodder maize crops [171]. The biopesticide formulation (A2085) original used strain is comparable to NRRL 21882 due to AF gene cluster lacking. Mytoolbox Af01 is another commercial product with the biocontrol strain, with a partial-cluster strain, which successfully reduces AFs in Serbia maize, due to the lack of AF cluster genes from *aflT* to *aflN* [172].

All new findings of these defected non-aflatoxigenic *A. flavus* isolates support an AF biocontrol strategy development to mitigate the AF content in crop. This new effective technology was first used widely in the US. Nowadays, this environmentally friendly and safe technology is annually employed over hundreds of thousands of hectares of susceptible crops [157,159,166,170].

Under the commercial name Aflasafe^TH^ (sorghum seed as a carrier of the non-aflatoxigenic strain of *A. flavus*) this improved biocontrol technology has been used in sub-Saharan Africa for more than 13 nations (Burkina Faso, Burundi, Gambia, Ghana, Kenya, Malawi, Mozambique, Nigeria, Rwanda, Senegal, Tanzania, Uganda, and Zambia), where effort to evolve biocontrol products is relevant, and it can be assumed that the number of participating nations will increase. Especially in African countries, modern technologies have been used to produce low-cost Aflasafe^TH^ products via mass production. Registered experimentally, Aflasafe^TH^ products have been proven to reduce AF levels in treated crops (e.g., maize and groundnut) by more than 80% compared to untreated crops with the same storage and field conditions [157,159,166,170].

### 5.2. The Selected Effective Post-Harvest Methods for Aflatoxin Mitigation

#### 5.2.1. Physical Post-Harvest Methods

##### Sorting

Most often, AFs contaminated grain is broken or damaged, which leads to inhomogeneous contamination of the entire volume of the stored crop; therefore, separation methods are suitable for decontamination [173]. Thus, sorting machines using the weight and size of particles as a parameter have been used for a long time. Airflow flotation and centrifugation used to be employed for sorting high volumes of grain, but sorting based on the optical principle was established in the 1960s. Due to the higher efficiency, this method is still used and is based on the principle of optical control when grains or peanuts are passing along the sensors. If a grain of a different color is detected, the magnetic valve opens and a thin stream of compressed air removes the grain [165]. For improvement of this currently used sorting method, for reduction of the risk of contamination, the single kernel sorting tool could be used for detection of multiple types of mycotoxins, including AFs, in peanuts and maize [174].

##### Dehulling

The precondition for the successful elimination of AF content is the restriction of colonization by AF-producing fungi on the surface layers of grains [165]. The outer layers of the grain are removed by dehulling techniques, which can remove up to 93% of the AFs [175].

##### Steeping

The first part of wet milling of maize grains, the steeping, consist of soaking the grains for 36–50 h at 50 °C in 0.1–0.2% SO_2_ water solution to disrupt protein matrix and improve germ separation and also induce production of lactic acid, which can be considered chemical treatment. The result is that the steeping liquor commonly obtains around half of the AF content [165,176]. Additionally, the level of AFs from sorghum grains could be decreased by steeping in 0.2% NaOH solution under levels of detection [177].

##### Wet Milling

Up to 40–50% of AFs could be eliminated from maize into the solution in wet milling; the remaining levels of AFs could be determined in the fiber fraction (28–38%), the gluten fraction (11–17%), germ (6–11%), and starch (1%) [165].

##### Dry Milling

Additional dry milling leads to the reduction of AF concentration in the germ fraction of the maize grain [165].

##### Heat Treatment

Temperatures above 160 °C have been shown to be effective in destroying pure AFB_1_, with soybean matrix accelerating the destruction process [178]. While temperatures up to 100 °C used for common food preparation have little effect on AFs, the higher temperatures used in frying, baking, roasting, and extruding may be more effective in reducing AF contamination [165].

AF levels can be decreased by extrusion by up to 50–80%, depending on the temperature and humidity of the grain, while the efficiency of the whole process can be increased by alkaline treatment. Additionally, in the case of peanut meal, extrusion alone leads to AF reduction by 23–66%, but coupled with ammonium hydroxide it can be up to 87%. Another heat treatment, roasting, leads to the reduction of AF amount in pecans and peanuts (50–70%) and maize (40–80%) [165].

##### Irradiation

Elimination of pathogenic organisms, and also, partially, AFs in food can be achieved by ionizing (gamma) or non-ionizing (solar, UV, microwave) radiation [165].

Compared with gamma-irradiation at 25 kGy (43% reduction) or microwave heating for 10 min (32% reduction), sunlight has been found to be more effective. Degradation of AFs by sunlight in cereals leads to reduction by 40% and up to 75% after 3 h and 30 h, respectively [179].

In other studies, gamma radiation has been used to irradiate maize, pistachio nuts, rice, and peanuts. The irradiation at 10 kGy induced 59–88% AF reduction. However, in another study, where irradiation at 15 kGy was employed, only 11–21% AF reduction has been proved [180,181].

The emission of UV-A (in dose 1200 mJ/cm^2^) has been shown to have a significant reduction effect on AFB_1_ and AFM_1_ in pure water by 70% and 84%, respectively. In cell culture studies, the increased dosage of UV- A emission has been shown to decreased or even suppress AF-induced cytotoxicity in HepG2 cells [182].

Pulsed Electric Fields

Due to AF thermostability, a pulsed electric fields (PEF) has been applied for its effective destruction. AFB_1_ and AF levels have been decreased by 77% and 97%, depending on the combination of different parameters as output voltage, pulse width, and pH, so the output voltage (20–65%), pH (4–10), and pulse width (10–26 µs), coupled with 2FI and quadratic models, result in PEF process optimization, which leads to AFB_1_ and total AF level reduction [183].

PEF treatment could be used for *Aspergillus parasiticus* inactivation and AF disintegration with alleviated mutagenic effects to preserve sesame seeds and their physicochemical properties. Levels of AFs B_1_, B_2_, G_1_, and G_2_, have been reduced by 86.9%, 98.7%, 94.7%, and, 92.7%, respectively with PEF energy in the range of 0.97 to 17.28 J, while the maximum PEF energy caused a 60% reduction of *A. parasiticus* [184].

#### 5.2.2. Chemical Post-Harvest Methods

Chemical post-harvest methods for AF mitigation are based on intervention with chemical agents, e.g., adsorbents, acids, and bases.

##### Adsorbents

Clay-based adsorbents have been proposed for use as a new technique for removing AFs from contaminated liquids [185]. A potential adsorbent may be, *inter alia*, bentonite [186], which is listed by United States regulations as a safe ingredient that can be used as a direct food ingredient for human [187]. The effect of bentonite in reducing AFM_1_ in milk has been demonstrated by several studies. AFM_1_ reduction has been observed when bentonite was added directly to naturally contaminated milk [188,189]. However, bentonite, when added to feed for dairy cattle, has also been found to be effective in reducing AFM_1_ levels in milk indirectly [190,191] via adsorption of AFB_1_ in the gastrointestinal tract, leading to reducing its carry-over as AFM_1_ into milk [192].

##### Acids

Another type of AF treatment using strong acids has been proven to be effective for the conversion of AFB_1_ and AFG_1_ to their hemiacetal forms, demonstrated in the case of HCl (pH 2), which decreased AFB_1_ concentration by 19% in 24 h [188]. Among other tested acids, e.g., citric, acetic, and lactic acids under simulating cooking conditions, the last one turned out to be the most effective in transformation of AFB_1_ and AFB_2_ [193].

##### Bases

AFs are unstable under alkaline conditions. Ammonization can reduce AF concentration by more than 99%. AF degradation by ammonia has been widely studied and has been shown to be effective in both laboratory and field experiments [165,194].

#### 5.2.3. Microbiological Post-Harvest Methods

Microbiological post-harvest methods for AF mitigation are based on intervention with microbiological agents as bacteria, and yeasts.

##### Bacteria

Lactic acid bacteria (e.g., *Bifidobacterium animalis* B subsp. *lactis*, *Enterococcus avium*, *Lactobacillus acidophilus*, *L. selangorensis*, *Lactococcus lactis* subsp. *lactis*, *Pediococcus acidilactici*, *Streptococcus thermophilus*, and *Weissella confuse*) have inhibitory effects on the AF production or cause the removal of AFs from foodstuffs and feedstuffs. The quality of AF binding by lactic acid bacteria strains depends on pH, temperature, the matrix itself, the incubation time, and also the inherent properties of the strain. AF elimination ranges for AFB_1_ from 16.3–98% and for AFM_1_ from 5.6–99.9%, and depends on the strain of lactic acid bacteria [163].

The degradation of AFs by probiotic bacteria to less or even non-toxic products has been shown to be an effective, safe, cheap, and environmentally friendly strategy of detoxification, with an approximately detoxification rate of 19–95% (for AFB_1_) and 12–100% (for AFM_1_) [106].

AF elimination by non-lactic acid bacteria (e.g., *Bacillus licheniformis*, *Bacillus stearothermophilus*, *Bacillus subtilis*, *Brachybacterium* spp., *Brevundimonas* spp., *Cellulosimicrobium funkei*, *Enterobacter* spp., *Escherichia coli*, *Mycolicibacterium fluoranthenivorans*, *Mycolicibacterium smegmatis*, *Myxococcus fulvus*, *Nocardia corynebacterioides*, *Pseudomonas aeruginosa*, *Pseudomonas stutzeri*, *Rhodococcus erythropolis*, *Streptomyces aureofaciens*, *Streptomyces lividans*, and *Stenotrophomonas maltophilia*) have an inhibitory effects on AF production or on the removal of AFs from milk at 4 °C. AF elimination ranges for AFB_1_ from 18–97% and for AFM_1_ from 32–64%, and depends on the strain of bacteria [163].

##### Yeast

In aflatoxigenic microfungi, the production of AFs has been significantly supressed by yeasts, e.g., *Candida*, *Debaryomyces*, *Pichia*, *Saccharomyces*, *Saccharomycopsis*, *Saccharomycodes*, *Schizosaccharomyces*, *Aureobasidium pullulans*, *Trichosporon*, and *Zygosaccharomyces*. AF elimination ranges for AFB_1_ from 15–100% and for AFM_1_ from 60–90.3%, and depends on the strain of yeasts [163].

It is known that yeast supplementation (e.g., *Pichia kudriavzevii* and *Kluyveromyces marxianus*) is able to detoxicate the AFB_1_ in rumina and reduce AFM_1_ levels in milk, which improves the dairy cattle performances [195].

#### 5.2.4. Genetic Engineering Post-Harvest Methods

Firstly, genetic engineering technologies are based on the regulation mechanism of AF biosynthesis in *A. flavus* that lack the ability to produce AFs. Only precise genomic integration of mutant allele methods is required for accurate understanding of the mechanism for regulation of AF biosynthesis produced by *A. flavus*. The new strategy for the foreign DNA site-specific integration in the *A. flavus sdh2* gene locus was evolved to prevent the disadvantage of ectopic or non-homologous recombination within integration of DNA into the genome [196].

Single substitution of amino acid (His 249 Leu) is involved in the mutant *sdh2^R^* allele on the pFC-eGFP vector, which has been established through cloning based on the yeast recombination for the transformation of fungi. *A. flavus* obtains systemic fungicide carboxin resistance as a result of a substitution of original *sdh2* allele with *sdh2^R^*. Proper integration of the *A. flavus* NRRL 3357 genome into the locus *sdh2* resulted in the highly efficient generation (>96%) of transformants [196]. The rapidity and effectiveness of this method consist of the locus *sdh2* with inserted eGFP expression cassette, which leads to the alleviation of virulence and the growth of fungi. This process would be a helpful instrument for genetic manipulation of *A. flavus* [196].

On the other hand, genetic engineering technologies are based on transforming maize plants to a transgenic AF-free cultivar employing host-induced gene silencing [197]. The evolved transgenic maize with a hairpin construct focused on transcription factor *aflR* of the AF biosynthesis was exposed to an aflatoxigenic strain of *A. flavus* originating from an endemic AF outbreaks in eastern Kenya. The results demonstrated that *A. flavus aflR* transcription factor colonizing transgenic maize was downregulated. Besides, transgenic maize kernels concentrated 14-fold lower AF levels in comparison with wild maize kernels. In the transgenic maize, the silencing cassette induced reduced kernel placement and its stunting, which probable led to ‘‘off-target’’ silencing of unintended genes by *aflR siRNAs* in transformed plants [197].

Another study revealed that host-induced gene silencing significantly eliminates the AF toxin from transgenic maize. The maize plants were transformed by using the gene cassette, with the kernel-specific RNA interference (RNAi), targeting the *aflC* gene, encoding the enzyme at the *Aspergillus* biosynthetic pathway of the AFs. In these kernels of transgenic maize, AFs have not been detected, compared to non-transgenic maize kernels, in which the levels of AF were in thousands of ppb after pathogen infection. Meanwhile, the same similarity between transcript developing groups of transgenic and non-transgenic kernels has been observed. It was proved that small interfering RNA molecules could be employed in maize AF biosynthesis silence, which could lead to its use as an attractive strategy, and in the view of food safety improvement, may be implemented in other crops [198].

Finally, there are genetic engineering technologies based on transformed peanut with genetic *A. flavus* infection resistance and AF production using host-induced gene silencing.

In the case of peanuts, the significant resistance method could be used through biosynthetic AF pathway genes (*aflM* and *aflP*) by host-induced gene silencing and by overexpressed plant defensins *MsDef1* and *MtDef4.2* with antifungal ability. The first method, in the case of AF infection, suppresses AF production to provide permanent resistance against various morphotypes of *A. flavus*, which results in insignificant levels of AFs in peanuts; the second one improves *A. flavus* infection genetic resistance. The significant relation between the accumulation of AFs and biosynthetic AF pathway gene transcription decrease has been confirmed in the case of overexpressed defensins as well as in host-induced gene silencing lines [199].

## 6. Summary

In 2020, it was 60 years since the discovery of AFs, which are, among all mycotoxins, considered to be the most agriculturally important and harmful. Some toxic effects of AFs have been observed, including carcinogenicity. The numerous effective pre-harvest and post-harvest biocontrol methods for AF mitigation have been applied. Research focused on AF genetic variability and the diversity of *A. flavus* and other producers of AFs is very important and solves biocontrol strategy problematics of non-aflatoxigenic A. *flavus* strains with a view toward better public health protection and to prevent economic losses. At Present, biocontrol strategies are sufficient; however, they should be further improved due to developing knowledge about recombination using transgenic A. flavus strains and the use of genome editing methods. Future research should be focused on elaborating these novel biocontrol strategies and their wide testing possibilities in ordinary foodstuffs and feedstuffs.

## Data Availability

Not applicable.
